# Role of Phenothiazines and Structurally Similar Compounds of Plant Origin in the Fight against Infections by Drug Resistant Bacteria

**DOI:** 10.3390/antibiotics2010058

**Published:** 2013-02-18

**Authors:** Sujata G. Dastidar, Jette E. Kristiansen, Joseph Molnar, Leonard Amaral

**Affiliations:** 1 Department of Microbiology, Herbicure Healthcare Bio-Herbal Foundation, 7&8 Metro Garden City, D. H. Road, Pailan Kolkata 700104, India; 2 Institute of Physics and Chemistry, University of Southern Denmark, Memphys, DK 5230, Odense, Denmark; 3 Department of Medical Microbiology and Immunology, University of Szeged, Dom ter 10, H-6720, Szeged, Hungary; 4 Travel Medicine of the CMDT, Institute of Hygiene & Tropical Medicine, Universidade Nova de Lisboa, 100 Rua Junqueira, 1349-008 Lisbon, Portugal

**Keywords:** phenothiazines, antimicrobial activities, efflux pumps, quorum sensing, reversal of antibiotic resistance

## Abstract

Phenothiazines have their primary effects on the plasma membranes of prokaryotes and eukaryotes. Among the components of the prokaryotic plasma membrane affected are efflux pumps, their energy sources and energy providing enzymes, such as ATPase, and genes that regulate and code for the permeability aspect of a bacterium. The response of multidrug and extensively drug resistant tuberculosis to phenothiazines shows an alternative therapy for the treatment of these dreaded diseases, which are claiming more and more lives every year throughout the world. Many phenothiazines have shown synergistic activity with several antibiotics thereby lowering the doses of antibiotics administered to patients suffering from specific bacterial infections. Trimeprazine is synergistic with trimethoprim. Flupenthixol (Fp) has been found to be synergistic with penicillin and chlorpromazine (CPZ); in addition, some antibiotics are also synergistic. Along with the antibacterial action described in this review, many phenothiazines possess plasmid curing activities, which render the bacterial carrier of the plasmid sensitive to antibiotics. Thus, simultaneous applications of a phenothiazine like TZ would not only act as an additional antibacterial agent but also would help to eliminate drug resistant plasmid from the infectious bacterial cells.

## 1. Introduction

Antibiotics have been found to be one of humankind’s most imperative weapons in combating microbial infections. Although there are highly effective antibiotics to cure nearly all major infectious diseases, such health benefits have come under threat, not only because many of these possess toxicity but also due to emergence of antibiotic-resistant bacteria. Therefore, the medicines required to cure major diseases threaten to erode the medical advances of recent decades. New antibacterial molecules and new therapeutic approaches are needed to overcome multi drug resistant (MDR) and extreme drug resistant (XDR) states in severe infectious diseases [1,2,3,4]. Thus, there is an indispensable need to explore newer molecules with lesser degrees of resistance [5]. Since the 1970s, several groups of workers independently undertook a systematic study to determine antimicrobial action of drugs belonging to various pharmacological classes not recognized as antimicrobials. This resulted in the accumulation of a large amount of evidence on many types of drugs possessing moderate to powerful antimicrobial action. All such drugs with antimicrobial activity are collectively termed as non-antibiotics (Kristiansen [6]). After the discovery by Paul Ehrlich [7] of the antimicrobial action of methylene blue, the search for drugs with antimicrobial property began. Ultimately the neuroleptic phenothiazine chlorpromazine (CPZ) was synthesized in 1950s. With global use of chlorpromazine, reports showed that patients receiving chlorpromazine had a lower incidence of bacterial infections [8]. After this, there was a boom in search for drugs, such as, antihistamines, anti-inflammatory agents, antihypertensives, cardiovascular drugs, antipsychotics and neuroleptics with possibilities of potent antimicrobial properties [9]. However, the antihistaminic and antipsychotic agents have been studied most extensively for their antimicrobial action both *in vitro* and *in vivo* [10,11,12,13]. 

## 2. Antimicrobial Action of Phenothiazines

Phenothiazines proved to be a unique class of compounds with prominent antibacterial activity against most of the pathogenic bacteria (Table 1). The MIC values of CPZ (chlorpromazine); Pr (promazine); Pz (promethazine); Pc, (prochlorperazine); Md (methdilazine); Fz (fluphenazine); Tm (trimeprazine); Tf (trifluoperazine); Tp (triflupromazine); Tz,(thioridazine); and Fp (flupenthixol). CPZ, Pr, Md, Fz, Tm, Tf, and Fp with respect to most of the Gram positive bacteria were from 10 µg/mL level, a few organisms could be inhibited by Md and Fz at 2 to 5 µg/mL level. The compound Tf was highly active against Gram positive bacteria as several of them revealed MIC as low as 2 µg/mL. Among Gram negative organisms, vibrios were most sensitive to many of the phenothiazines. However, several strains of *Salmonella* spp. and *Shigella* spp. exhibit greater sensitivity than others of the same genera. Klebsiellae, pseudomonads and acenetobacters were highly resistant to almost all of these drugs. Many of these phenothiazines were bacteriostatic, while some others were able to kill a pathogen within 6 to 18 h. 

**Table 1:** Antibacterial activity of synthetic phenothiazines by *in vitro* screening.

Phenothiazine	MIC µg/mL		Bacteriostatic/ Bactericidal
	Gram +ve	Gram –ve	
CPZ	10–50	25–100	Bacteriostatic for Gm −ve, Bactericidal for Gm +ve
Pr	10–50	10–100	Bacteriostatic
Pz	50–200	100–200	Bacteriostatic
Pc	25–100	50–400	Bacteriostatic
Md	10–100	25–200	Bactericidal
Fz	10–100	10–100	Bactericidal
Tm	10–100	10–100	Bactericidal
Tf	10–100	25–200	Bactericidal
Tp	2–50	2–100	Bactericidal
Tz	32–64	100–800	Bacteriostatic for Gm –ve, Bactericidal for Gm +ve
	50–800		
Fp	5–50	10–100	Bacteriostatic

**Please note: The bactericidal effect can be reached with multiple of the MICs. **CPZ, chlorpromazine; Pr, promazine; Pz, promethazine; Pc, prochlorperazine; Md, methdilazine; Fz, fluphenazine; Tm, trimeprazine; Tf, trifluoperazine; Tp, triflupromazine; Tz, thioridazine; Fp, flupenthixol.

The mechanism by which the phenothiazines act on bacterial cells *in vitro* has been studied by several researchers during the past few years. In 1979, Kristiansen [14] observed that CPZ was bacteriostatic to *S. aureus* at low level, but as the doses of CPZ were increased, it produced bactericidal action on the same organism. It was shown further that CPZ was involved in bacterial haemolysins, as the erythrocytic membranes of animals were altered in such way that haemolysis of the membrane was affected. Therefore, at low concentrations CPZ possibly interfered with the transport of potassium through the bacterial membrane much in the same way as it is occurs in mammalian tissue [14]. In 1986, Galeazzi *et al* [15] observed that CPZ was a competent cell permeabilizer and was capable of conducting microbial peroxidase and peroxidase like reactions. Whenever studied, CPZ increases the permeability of the bacterium to antibiotics, as evident from the items presented in this herein review. 

In 1991, Amaral and Lorian [16] observed that when *E. coli* was grown at the sub-MIC level of CPZ, the cells became elongated and filament-like in 5 h, but reverted to rod-like shape after 24 h. It was found that the electrophoretic pattern of proteins extracted from the cell envelopes of all forms of CPZ treated cells was distinctly different from those of both the untreated cells of *E. coli*.

In 2000, Amaral *et al* [17] observed that CPZ failed to produce any inhibitory effect on cell proliferation of *Salmonella* that were allowed to remain in the sub-inhibitory state of agglutinability with the specific O antibody. Thus, the resistance to CPZ was dependent upon changes induced by CPZ in the cell wall. It was postulated that CPZ probably was able to bind with 55 KDa protein in the cell wall and interfered with the recognition of O antigen by the specific antibody.

According to Radhakrisnan *et al* [18], phenothiazine thioridazine (TZ) proved to be a unique drug, as it could induce complete destruction of different Gram positive bacteria within a span of only two hours; however, with respect to all the different Gram negative organisms it was observed that although there was a gradual decrease in the number of viable cells after addition of Tz in a highly multiplying state of the organisms, the cells remained viable up to 18 h, revealing the bacteriostatic nature of Tz on such bacteria. It was suggested that the drug was possibly able to penetrate quite easily the peptidoglycan layer of the cell wall of Gram positive bacteria, but was unable to have any negative effect on the components of the outer membrane of Gram negative cell envelope such as lipoprotein or the lipopolysaccharide.

Since there is no specific drug to cure the sleeping sickness caused by *Trypanosoma brucci*, Page and Lagando [19] investigated the action of Tz on the pellicular membrane complex of the infective bloodstream form of the parasite. Although Tz could induce rapid changes in cell shape but failed to affect structural integrity of the microtubular complex. However, the drug was successful in damaging both the nuclear and the cytoplasmic membranes. In this way like CPZ Tz was also found to have action on cell envelopes. 

Investigations on the structure activity relationships of the phenothiazines containing halogen atoms showed that their antimicrobial properties were possibly linked to the methyl-thio substituent at position 10 and a halogen moiety at position 2 of the basic phenothiazine ring [20]. As the thioxanthene skeleton is similar to phenothiazine except for the absence of a tertiary nitrogen atom at position 9, the presence of a trifluoromethyl group at position 2 of the tricyclic ring may be possible for rendering the antibacterial property to Fp, producing a structure similar to the anti-inflammatory antibacterial agent diclofenac sodium [10]. All these studies revealed that both CPZ and Tz have different kinds of action on the cell envelopes of both Gram positive and Gram negative bacteria.

To evaluate the efficacy of phenothiazines in animal systems, a series of studies were conducted with a Swiss strain of male white mice weighing 18–20 g each were taken (Table 2). The naturally mouse virulent bacterium *Salmonella enterica* serovar Typhimurium NCTC 11 and NCTC 74 obtained from London served as the challenge strains. Both these strains were simultaneously sensitive to many antibiotics and the phenothiazines. Virulence of strains was significantly increased with repeated mouse passages and the median lethal dose (MLD or LD50) was determined following standard technique [21]. Protective capacity of each phenthiazine was determined by injecting a definite dose of the drug followed by challenge with 50 LD_50_ dose of the virulent salmonella to groups of mice. Toxicity levels of the compound were determined at the same time. In a separate experiment, the actual bacterial load in various organs was determined in treated and untreated mice. While evaluating the effects of phenothiazines in challenged mice it was noted that Pr was the best drug since it could offer protection at the level of 2–8 µg/mouse, and Tm was the next in order. However, Pr was much less toxic than Tm since the latter produced severe convulsion followed subsequently by death when the doses were greater than 16 µg/mouse. The drugs Md, Tf, Tp and Fp were much less toxic and offered statistically significant protection at the levels of 15–30 µg/mouse. Since the *in vitro* MIC of Tz in *Salmonella enterica* 74 was 500 µg/mL 200 µg/mouse was required to protect the challenged mice. Higher amounts of Tz also produced convulsion in animals (Table 2).

**Table 2:** Anti-salmonella activity of phenothiazines *in vivo.*

Phenothiazine	Drug (µg/g) per mouse	
	Toxic dose	Protective dose
Pr	>64	2–8
Md	>320	15–30
Fz	>120	30–60
Tm	>16	4–8
Tf	>60	15–30
Tf	>60	15–30
Tz	>500	200
Fp	>60	15–30

Pr, promazine; Md, methdilazine; Fz, fluphenazine; Tm, trimeprazine; Tf, trifluoperazine; Tp, triflupromazine; Tz, thioridazine; Fp, flupenthixol.

It is known that phenothiazines are concentrated by macrophages almost up to 100-fold of its original amount in a medium in which macrophages are maintained in the laboratory [22,23]. These increases of intracellular concentration take place in the lysosome [13,22,23] resulting in reaching the bactericidal level of the compound [22,23,24]. According to Amaral *et al* [17] a phenothiazine may promote loss of 55 KDa virulence protein and hence there is a great possibility that viable cells of salmonella lose their virulence inside the phagolysozome. Although a very large number of viable cells of *S. enterica* are retrieved from untreated animals 18h after challenge, there was always statistically significant reduction in the number of viable cells recovered from treated animals. From such data, however, a definite conclusion cannot be made regarding loss of virulence proteins in the phagocytosed salmonellae until the exact mechanism is unveiled and determined. Nevertheless, it is now known that a phenothiazine such as TZ affects the activity of genes that play a role in the survival of the Gram-negative bacterium [24,25]. The main genes affected by exposure to a phenothiazine such as thioridazine are those that code for plasma membrane based proteins that regulate the permeability of the cell envelope [25]. 

## 3. Antimicrobial Action Phenothiazine-Like Compounds from Plants

In a study of determination of antimicrobial potentiality of different plant extracts Dastidar *et al.* [26] observed that a prenylflavonone labeled as YS06 procured from the root of *Sophora* plant was active both *in vitro and in vivo* (Table 3). The *in vitro* MIC values were between 25 and 200 µg/mL level of the pure compound; it was bactericidal and could ably protect mice infected with *S. enterica* at doses of 40–80 µg/mouse. Such a phenomenon was further confirmed by determining reduction in the number of viable cells in mice receiving both prenylflavonone and the challenge when compared to the set of animals that were given the challenge only. Subsequently an isoflavonoid compound (YS19) derived from the same plant revealed that this was a bacteriostatic agent and could inhibit bacterial growth at 25–200 µg/mL level and successfully protected mice at an amount of 30–60 µg/mouse [27]. Subsequent animal experiments showed that much like YS06, YS19 could also reduce number of viable salmonellae in spleen, liver and heart blood of mice receiving both the agent and organism.

**Table 3:** Antibacterial action of plant derived compounds.

Compound of plant origin	MIC (µg/mL)		Type of action	Animal protection dose / mouse
	Gm +ve	Gm –ve		
Prenylflavonone YS06	25–100	25–100	Bactericidal	40–80 µg
Isoflavonoid YS19	25–200	25–200	Bacteristatic	30–60 µg
*Mesua ferrea* flower extract		50–100	Bactericidal	50–100 µg
Flavonone from *Butea frondosa* bark	50–200	50–200	Bacteristatic	50–200 µg

Mazumder *et al*. [28] observed that the flower extract of *M. ferrea* possessed potent *in vitro* bactericidal action on salmonellae, and that the extract was able to offer significant protection to mice challenged with virulent salmonellae. In 2008, Mishra *et al*. isolated a flavonone from the bark of *Butea frondosa* and detected powerful antibacterial action both *in vitro* and *in vivo*. Many other antimicrobial compounds have been isolated [29,30,31,32,33]. Thus microorganisms are not the only source of antibacterial agents like antibiotics, but various other studies further strengthen the possibilities of procuring and securing from many types of natural sources.

## 4. Special Aspects and Activities of Phenothiazines

The majority of medicinal compounds in use today owe their origin to a given phenothiazine [34]. This is not surprising since these compounds have activities on the plasma membrane of bacteria [35,36,37], protozoa [38], eukaryotes [39]; in short, all living cells. The following sections discuss specific aspects of phenothiazine activities inasmuch as these activities have potential for the development of new medicinal compounds for therapy of infections and cancers. The reader is encouraged to visit reference 32 for a comprehensive presentation of the evolution of phenothiazines as antimicrobial agents.

### Phenothiazines and the Plasma Membrane

In general, phenothiazines are electron donors and bind by charge transfer complexes (CTC) formation to target molecules when an electron is supposed to go from the highest filled molecular orbital to the lowest empty orbital of the acceptor molecule on the target. When the phenothiazine acts as an electron donor at the surface of the plasma membrane of the cell or within the lipid bilayer of the plasma membrane, then the electron transfer on the outside will result in depolarization of the membrane. Because this depolarization reduces the activity of the plasma membrane (conductivity, *etc.*), the phenothiazine has been referred to as a membrane-stabilizing agent. However, when the phenothazine acts as an electron donor on the cytoplasmic side of the plasma membrane, hyperpolarization results and membrane-linked processes are inhibited. If the biological activity is actually due to charge transfer complex formation, we expect pharmacological activity from electron donation by the phenothiazine (there are some exceptions to this rule: CPZ- sulfon- or, sulphoxydes and methylene blue, where due to the asymmetric distribution of charge distribution main cause for ineffective activity). In general, one may say that the activity of the phenothiazine on the medial side of the plasma membrane is dependent upon a very high concentration of the compound. These concentrations are clinically irrelevant since they cannot be safely achieved in the patient but can be readily achieved *in vitro.* Therapeutically, a phenothiazine such as CPZ is administered at far lower concentrations that limit the activity of the agent to the surface of the plasma membrane (*i.e.*, electron acceptor). It should be noted that a variety of agents can obviate the surface activity of CPZ such as caffeine [40]. *In vitro* caffeine forms precipitates with CPZ therefore reducing the neuroleptic effects of the agent. At the level of the plasma membrane they can disperse CPZ from its binding sites of the neuron hence patients who are managed with CPZ must take care not drink excessively caffeine rich liquids such as tea and coffee.

The main mechanism of action of most phenothiazines that have a variety of effects on the activity of the plasma membrane involves the inhibition of calcium binding to calcium dependent enzymes [41]. However, because of the differentiation of cells, the constituents on the surface of the plasma membrane determine whether a specific phenothiazine will have activity on that given cell type [42]. This means that various members of the phenothiazine group may present with specific activities; e.g., neuroleptics chlorpromazine and flupentazine and the phenothiazine derived antihistamines methdilazine and trimeprazine the tranquilliser promethazine. Nevertheless, although the major mechanisms may differ, whenever studied, most phenothiazines have activity against bacteria albeit at *in vitro* concentrations which are clinically irrelevant 

Among the activities reported for phenothiazines are those that affect the activity of efflux pumps of bacteria, mycobacteria and cancer cells that express a multi-drug resistant phenotype [43]. Efflux pumps extrude noxious agents that penetrate into the cell and therefore afford protection from those agents. To the bacterium or cancer cell, antibiotics and anticancer agents are noxious agents that must be expelled prior to reaching their intended targets. Although all living cells have these efflux pumps at a basal level, they can be rapidly over-expressed when the concentration of an agent is increased [44,45,46,47,48]. Moreover, other proteins that regulate permeability of the cell envelope such as porins, are down-regulated [45,46].

With respect to bacteria, serial exposure to increasing concentrations of an antibiotic results in progressive increases in resistance to the given antibiotic. Serial exposure of pansusceptible *Mycobacterium tuberculosis* to progressive increases of isoniazid (INH) increases resistance to the drug [49]. Similar exposure of antibiotic susceptible *Escherichia coli* to increasing concentrations of tetracycline promote progressive increases of resistance to the antibiotic that is accompanied by increased expression of genes that regulate and code for various efflux pumps of the organism [50]. If at any one point during the latter study the last concentration of tetracycline is serially maintained, further increases in the expression of efflux pump genes takes place and accompanied with accumulation of mutations in genes that code for proteins sensitive to beta-lactams, streptomycin and gyrase A. As prolonged exposure to a constant concentration of tetracycline, the expression of efflux pump genes is reduced to base-line levels [51,52]. These results have been interpreted to indicate that the organism follows the 2nd law of thermodynamics inasmuch as the energy needed for maintenance of an over-expressed efflux pump system is great, and given the unchanging environment containing a high level of the noxious agent (antibiotic), it can conserve energy by activating a mutator gene that promotes mutations in essential proteins, as predicted by Chopra *et al.* [53]. In all studies so far conducted, including those involving other Gram-negatives [25] and Gram-positives [54,55,56] and mycobacteria [49,57,58,59] phenothiazines such as chlorpromazine and thioridazine reverse the antibiotic induced resistance. 

The mechanism by which a phenothiazine reverses efflux pump mediated resistance to an antibiotic appears to be indirect. Firstly, depending on the environmental pH, the source of energy that drives the efflux pump differs. At pH lower than 7, the phenothiazine does not inhibit the efflux of a noxious agent whereas at pH above 7, inhibition of efflux results from exposure to a concentration of the phenothiazine that is devoid of antibacterial activity [60]. These results are interpreted to indicate the possibility that the phenothiazine inhibits the generation of hydronium ions from the hydrolysis of ATP by ATP synthase activity, and therefore, the maintenance of the proton motive force is affected. Because at low pH of the environment, the hydronium ions that are bound at the surface of the cell envelop [61,62] create the proton motive force, the needed energy for efflux is independent of metabolism and therefore not affected by then phenothiazine. Lastly, phenothiazines are well known inhibitors of the proton motive force at pH *ca*. 7 [63,64], therefore the interpretation of the pH dependent effects of the phenothiazine on the efflux pump of bacteria receives support.

## 5. Therapy of MDR/XDR/TDR TB

Since the 1950s, as a consequence of extensive use of chlorpromazine for therapy of psychosis, sporadic reports appeared suggesting that this neuroleptic could cure a pulmonary tuberculosis infection [8]. However, it was the advent of multi-drug resistance world-wide during the late 1980’s that the use of chlorpromazine for therapy of tuberculosis was seriously considered and immediately dismissed due to the severe toxicity produced by this neuroleptic. Moreover, the concentrations of chlorpromazine needed were in the range of 15 to 30 mg/L, and this was far greater than that which could be safely achieved in the patient (maximum plasma concentration clinically achieved safely is *ca*. 0.5 mg/L). Nevertheless, interest in chlorpromazine as an anti-tubercular drug continued and when Crowle and his group [65] showed that clinically relevant concentrations of chlpromazine in the medium could promote the killing of intracellular *Mycobacterium tuberculosis* [65], interest in this agent was increased. Soon thereafter, the milder neuroleptic thioridazine was shown to have activity against all encountered antibiotic resistant strains of *Mycobacterium tuberculosis* (mono-resistant; multi-drug resistant and extensively drug resistant strains of *Mycobacterium tuberculosis*) [66]. Later studies demonstrated that thioridazine promoted the killing of intracellular multi-drug resistant [67] and extensively drug resistant strains [68] of *Mycobacterium tuberculosis* and could cure the mouse infected with antibiotic susceptible [69] and multi-drug resistant [70] strains of *Mycobacterium tuberculosis.*

These studies laid down the foundation for the first demonstration that thioridazine in combination with antibiotics to which the *Mycobacterium tuberculosis* was initially resistant, could cure rather quickly, patients infected with extensively drug resistant *Mycobacterium tuberculosis* [71]. The mechanism by which these cures have been achieved involves the activation of lysosomal hydrolases resulting from the inhibition of potassium efflux from this organelle [72,73,74] and by inhibition of the source of energy needed for adequate function of the efflux pump system that afforded a multi-drug resistant phenotype of the infecting organism [72,73,74]. It should also be mentioned that the use of thioridazine as monotherapy of the extensively drug resistant tuberculosis patient results in rapid improvement in the quality of life in that the patients regain their appetite, put on weight, night sweats are reduced and even obviated, and because of the neuroleptic activity of thioridazine, stress that results from this infection is markedly reduced [75]. These latter studies have been expanded by Utwadia *et al.* [76], and as a result, thioridazine has been recommended for use as a “salvage drug” for therapy of the extensively drug resistant TB patient [75].

## 6. Concluding Remarks

Antipsychotics block D_2_ receptors in the dopamine pathway of brain such that dopamine released in this pathway has a lesser effect. The tricyclic compound phenothiazines are used as antidepressant and anxiolytic and antipsychotic agents. They accumulate in the brain provoking blockade of dopamine receptors inasmuch as excess release of dopamine in the mesolimbic pathway has been linked to psychotic experiences. High potency antipsychotic drug like haloperidol can be applied in doses of a few milligrams causing sleepiness and a calming effect in patients within minutes, while low potency antipsychotics like CPZ or TZ require doses of several hundred milligrams to produce the same action. These have a much greater anticholinergic and antihistaminic actions that can counteract dopamine related side effects. Most of the antimicrobial phenothiazines are of this order. 

Phenothiazines have their primary effects on the plasma membranes of prokaryotes and eukaryotes. Among the components of the prokaryotic plasma membrane affected are efflux pumps, their energy sources, energy providing enzymes, such as ATPase and genes that regulate and code for the permeability aspect of a bacterium. The response of multidrug and extensively drug resistant tuberculosis to phenothiazines shows an alternative therapy for treatment of these dreaded disease that is claiming more and more lives every year throughout the world. Many phenothiazines have shown synergistic activity with several antibiotics thereby lowering the doses of antibiotics administered to patients suffering from specific bacterial infections. Trimeprazine is synergistic with trimethoprim [77]. Fp has been found to be synergistic with penicillin [78] and CPZ plus some antibiotics are also synergistic [16].

Along with antibacterial action described in this review, many phenothiazines possess plasmid curing activities, which render the bacterial carrier of the plasmid sensitive to antibiotics [55,77,78,79,80,81]. Thus simultaneous applications of a phenothiazine like TZ would not only act as an additional antibacterial agent but also would help to eliminate drug resistant plasmid from the infectious bacterial cells.
